# Recent Advances in Our Understanding of the Biosynthesis of Sulfur Modifications in tRNAs

**DOI:** 10.3389/fmicb.2018.02679

**Published:** 2018-11-01

**Authors:** Naoki Shigi

**Affiliations:** Biotechnology Research Institute for Drug Discovery, National Institute of Advanced Industrial Science and Technology (AIST), Tokyo, Japan

**Keywords:** biosynthesis, iron-sulfur cluster, post-transcriptional modification, radical SAM enzyme, sulfurtransferase, sulfur modification, tRNA

## Abstract

Sulfur is an essential element in all living organisms. In tRNA molecules, there are many sulfur-containing nucleosides, introduced post-transcriptionally, that function to ensure proper codon recognition or stabilization of tRNA structure, thereby enabling accurate and efficient translation. The biosynthesis of tRNA sulfur modifications involves unique sulfur trafficking systems that are closely related to cellular sulfur metabolism, and “modification enzymes” that incorporate sulfur atoms into tRNA. Herein, recent biochemical and structural characterization of the biosynthesis of sulfur modifications in tRNA is reviewed, with special emphasis on the reaction mechanisms of modification enzymes. It was recently revealed that TtuA/Ncs6-type 2-thiouridylases from thermophilic bacteria/archaea/eukaryotes are oxygen-sensitive iron-sulfur proteins that utilize a quite different mechanism from other 2-thiouridylase subtypes lacking iron-sulfur clusters such as bacterial MnmA. The various reaction mechanisms of RNA sulfurtransferases are also discussed, including tRNA methylthiotransferase MiaB (a radical *S*-adenosylmethionine-type iron-sulfur enzyme) and other sulfurtransferases involved in both primary and secondary sulfur-containing metabolites.

## Introduction

**FIGURE 1:**
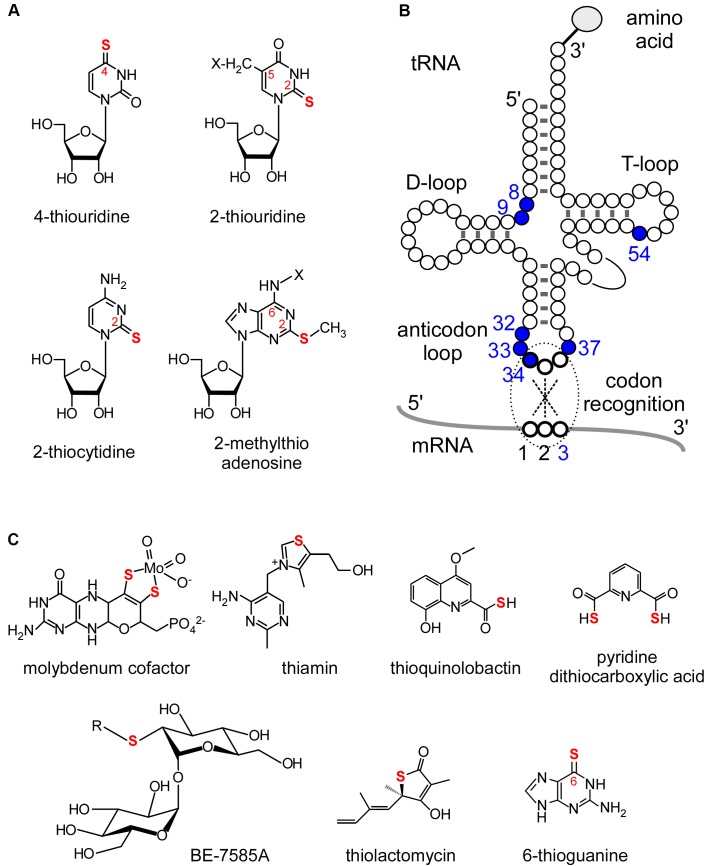
Sulfur modifications in tRNAs and other sulfur-containing metabolites. **(A)** Chemical structures of sulfur modifications in tRNAs. **(B)** Positions of thionucleosides in tRNAs. **(C)** Examples of sulfur-containing cofactors and secondary metabolites.

Transfer RNA (tRNA) is an essential adaptor molecule that bridges genomic information from mRNAs to amino acid sequences in proteins. Precursor tRNA molecules undergo various maturation steps such as removal of leader, trailer, and intronic sequences, addition of 3′-CCA sequences, and chemical modification of nucleosides. More than 100 post-transcriptional modifications of tRNAs have been identified (Cantara et al., 2011; Väre et al., 2017; Boccaletto et al., 2018), among which sulfur modifications are especially important for tRNA functions. Four kinds of thionucleoside derivatives are found in tRNAs (Figures 1A,B): 4-thiouridine (s^4^U) at positions 8 and 9 (Lipsett, 1965; Singer and Smith, 1972; Griffey et al., 1986), 2-thiocytidine (s^2^C) at position 32 (Carbon et al., 1968; Murao et al., 1972), 2-thiouridine (s^2^U) at position 33 (Crain et al., 2002), 2-thiouridine derivatives (xm^5^s^2^U) at positions 34 (Carbon et al., 1968; Oashi et al., 1970) and 54 (Watanabe et al., 1974), and 2-methylthioadenosine derivatives (ms^2^x^6^A) at position 37 (Burrows et al., 1968; Ishikura et al., 1971) (where “x” represents several functional groups differing between species and organelles). At position 34, there is taurine (2-aminoethansulfonic acid)-containing modification at C5 carbon of U (Suzuki et al., 2002), 2-selenouridine derivatives (xm^5^se^2^U), and 2-geranyl-thiourideine derivatives (xm^5^ges^2^U) (Wittwer et al., 1984; Dumelin et al., 2012). The biosynthesis of tRNA sulfur modifications involves sulfur trafficking systems and “modification enzymes.” The sulfur trafficking systems used in RNA modification are closely related to and shared with cellular sulfur metabolism (Laxman et al., 2013), whereas modification enzymes recognize substrate tRNAs and incorporate sulfur atoms. Some sulfur-containing cofactors and secondary metabolites are depicted in Figure 1C.

## Functions of Sulfur Modifications in Trnas

The functions of sulfur modifications are briefly summarized in this section. For more detailed information, please refer to previous reviews (Shigi, 2014, 2016) and articles cited therein. Uridine at position 34 (the wobble base) of tRNAs for lysine, glutamic acid, and glutamine is almost universally modified to s^2^U derivatives, although the C5 carbon of uridine is also modified by functional groups that differ between species (Elseviers et al., 1984). Due to steric clashes between the bulky 2-thio group and the 2′-OH group of ribose, the ribose of s^2^U preferentially adopts the C3′-*endo* conformation (Yokoyama et al., 1985; Agris et al., 1992). Therefore, xm^5^s^2^U stabilizes base paring with NNA and NNG codons for lysine, glutamic acid, and glutamine (Agris et al., 1973; Murphy et al., 2004; Durant et al., 2005; Johansson et al., 2008). Absence of the 2-thio modification leads to ribosome stalling at AAA, CAA, and GAA codons in mRNAs. Interestingly, pausing of the ribosome causes protein misfolding and aggregation (Nedialkova and Leidel, 2015), suggesting that optimal codon translation by tRNA wobble modifications is very important for maintaining proteome integrity. tRNA modifications are proposed to control the translation efficiency of specific groups of genes with mRNA codon bias as a mechanism of adaptation to specific environments (Laxman et al., 2013; Tigano et al., 2015; Tyagi and Pedrioli, 2015; Chionh et al., 2016).

In addition to position 34, the distribution of sulfur modifications at other positions differs between species, and modifications at positions 32 and 37 in the anticodon loop are also important for precise codon recognition. The 2-methylthio modification at position 37 directly stabilizes mRNA-tRNA interactions with U in the anticodon third position and A in the codon first position, as revealed by structural analysis of mRNA-tRNA interactions in the ribosome (Jenner et al., 2010). The s^4^U modification at position 8 is responsible for near-ultraviolet light sensing in bacteria (Favre et al., 1969; Carre et al., 1974; Ryals et al., 1982). When the cell is irradiated with near-ultraviolet light, s^4^U crosslinks with cytidine 13, resulting in a disordered tRNA structure that leads to translational arrest. In some thermophilic microorganisms, such as *Thermus thermophilus* and *Pyrococcus furiosus*, 5-methyl-2-thiouridine (m^5^s^2^U) or 2-thioribothymidine (s^2^rT) is found at position 54 in almost all tRNA molecules (Watanabe et al., 1974; Kowalak et al., 1994), and the 2-thiolation content increases with increasing cultivation temperature (Watanabe et al., 1976; Kowalak et al., 1994). The m^5^s^2^U modification strengthens the duplex structure formed by the D-loop and T-loop, which stabilizes the overall tRNA structure (Horie et al., 1985). In *T. thermophilus*, m^5^s^2^U is indispensable for growth at high temperature (Shigi et al., 2006a). s^2^U at position 33 was also found in mitochondrial tRNA^Trp^ from *Leishmania* (Crain et al., 2002), the function of this modification has not been elucidated.

## Biosynthetic Pathways for Sulfur Modifications in Trnas

In eukaryotes and bacteria, sulfur atoms in sulfur-containing molecules such as thionucleosides are derived from free L-cysteine in the cell. The sulfur atom of L-cysteine is activated by cysteine desulfurase, a pyridoxal-5′-phosphate (PLP)-dependent enzyme, via covalent attachment to its catalytic cysteine residue to generate the persulfide (R-SSH) form (Flint, 1996; Lauhon and Kambampati, 2000; Lauhon, 2002; Nilsson et al., 2002). Enzyme-linked persulfides are then transferred to downstream sulfur carrier proteins, and eventually transferred to the final sulfurtransferases in each pathway (Mueller, 2006; Shi et al., 2010). Thus, biosynthetic pathways for thionucleosides are part of a larger metabolic system involving other sulfur-containing molecules with iron-sulfur (Fe-S) clusters, thiamin, and the molybdenum cofactor (Moco) (Figure 1C; Schindelin et al., 2001; Settembre et al., 2003; Hidese et al., 2011). Moreover, each pathway is mutually influenced by others as part of a “sulfur trafficking” network (Maynard et al., 2012; Dahl et al., 2013). Other fascinating features include the involvement of numerous sulfur carrier proteins that deliver activated sulfur species such as R-SSH and thiocarboxylates (R-COSH), and the mechanisms by which they achieve the safe, directional flow of potentially harmful sulfur atoms (see below).

Thionucleoside synthesis can be classified into two types based on the involvement of Fe-S proteins, and hence the dependency on Fe-S cluster biosynthesis. The biosynthesis of s^2^C32, ms^2^A37, and m^5^s^2^U54 is dependent on Fe-S clusters (Lauhon et al., 2004; Leipuviene et al., 2004; Chen et al., 2017). Biosynthesis of s^4^U8 and s^2^U34 differs among species; the biosynthesis of s^4^U8 in bacteria, such as *E. coli*, *Salmonella typhimurium, Bacillus subtilis* (Lauhon et al., 2004; Leipuviene et al., 2004; Rajakovich et al., 2012), and some archaea, such as *Thermoproteales*, *Thermoplasmatales*, *Halobacteriales*, and *Sulfolobales* (Liu et al., 2012), is not dependent on Fe-S clusters, while in methanogenic archaea and some other archaea, such as *Thermococcales*, it is dependent on Fe-S clusters (Liu et al., 2012, 2016). The biosynthesis of s^2^U34 is not dependent on Fe-S clusters in bacteria (Lauhon et al., 2004; Leipuviene et al., 2004; Black and Dos Santos, 2015), but the opposite is true for archaeal and eukaryotic pathways (Nakai et al., 2007; Liu et al., 2016).

se^2^U34 is synthesized from s^2^U34 via geranylated intermediate (ges^2^U) by SelU (YbbB) (Chen et al., 2005; Dumelin et al., 2012; Sierant et al., 2018b). Desulfuration activity of 2-thiouracil by DUF523 domain protein in the cell has recently been reported (Aucynaite et al., 2018). Extensive *in vitro* analysis of desulfuration of s^2^U derivative as a form of nucleoside or within an RNA chain has been performed, hydrogen peroxide and cytochrome C or Fe^II^ -mediated reactions forms predominantly generate 4-pyrimidinone nucleoside (h^2^U), rather than U (Sochacka et al., 2013; Sierant et al., 2018a). These studies will lead to better understanding of the *in vivo* metabolism of thionucleosides.

## The Role of Sulfurtransferase Mnma in 2-Thio U Synthesis

**FIGURE 2:**
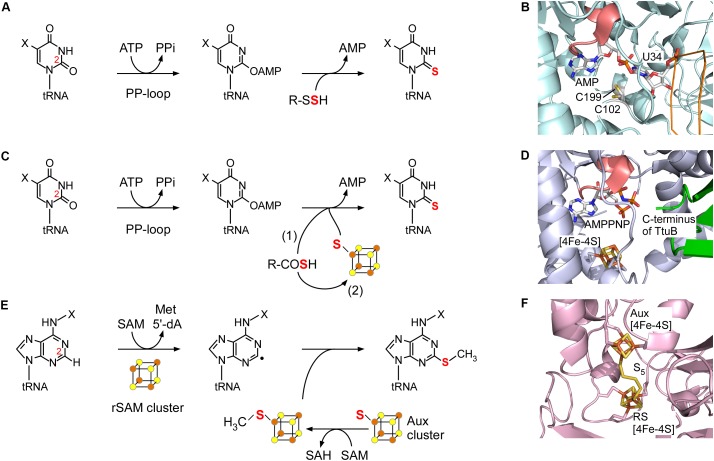
Two-step reaction mechanism of representative (methyl)sulfurtransferases. **(A)** The 2-thiouridylation reaction catalyzed by MnmA. A protein persulfide is utilized as the sulfur donor. **(B)** Crystal structure of *Escherichia coli* MnmA, showing the tRNA complex with an adenylated intermediate (PDB ID: 2deu). The backbone of the tRNA and the PP-loop of MnmA are colored orange and pink, respectively. The two catalytic cysteines (Cys102 and Cys199) are shown in stick representation. **(C)** The 2-thiouridylation reaction catalyzed by TtuA/Ncs6. A protein thiocarboxylate may be utilized directly as shown in scheme (1). Alternatively, a free sulfide from the thiocarboxylate may be utilized once trapped by the Fe-S cluster as shown in scheme (2). **(D)** Crystal structure of the *Thermus thermophilus* TtuA (blue)-TtuB (green) complex (modeled from PDB ID: 5b4e and 5gha). The ATP analog bound to the PP-loop (pink), and the C-terminus of TtuB and the Fe-S cluster encounter one another in the catalytic center of TtuA. Note that two residues of the C-terminus of TtuB are not visible. **(E)** The 2-methylthioadenylation reaction catalyzed by MiaB. A methylsufide formed on the auxiliary (Aux) cluster is utilized as a substrate. **(F)** Crystal structure of *Thermotoga maritima* RimO clearly showing the pentasulfide bridging the rSAM and Aux clusters (PDB ID: 4jc0).

MnmA is a thiouridylase that catalyzes 2-thiolation of uridine at position 34 in bacteria (Kambampati and Lauhon, 2003). The homologous enzyme Mtu1 is involved in s^2^U formation in eukaryotic mitochondria (Umeda et al., 2005). In *Escherichia coli*, TusA, the TusBCD complex, and TusE are required for s^2^U formation (Ikeuchi et al., 2006). TusA interacts with cysteine desulfurase IscS, accepts the persulfide, and directs sulfur flow to this pathway. MnmA accepts the persulfide sulfur on its conserved Cys199 residue in the active site from TusA via TusD and TusE. However, in most species, there is no need for such intermediate persulfide carrier proteins (Black and Dos Santos, 2015). MnmA possesses a PP-loop motif and is a member of the ATP-pyrophosphatase family. This enzyme utilizes a two-step mechanism to form an adenylated intermediate (Figure 2A). Nucleophilic attack by the persulfide sulfur generates s^2^U and releases AMP. The modification enzyme ThiI involved in s^4^U synthesis also contains a PP-loop and utilizes a similar two-step mechanism (Mueller et al., 1998; Mueller and Palenchar, 1999; Neumann et al., 2014). A snapshot of s^2^U formation via the acyl-adenylated intermediate was clearly revealed in a structural analysis of the *E. coli* MnmA-tRNA complex (Figure 2B; Numata et al., 2006). In the catalytic pocket, which is separated from bulk solvent, the uridine reacts with ATP to form an acyl-adenylated intermediate that reacts with the terminal sulfur released from the persulfide on Cys199 with assistance from another conserved cysteine (Cys102).

## The Role of Iron-Sulfur Protein Ncs6/Ttua in 2-Thio U Synthesis

In eukaryotes and archaea, Ncs6 and its archaeal homolog NcsA catalyze the 2-thiolation reaction of uridine at position 34 (Bjork et al., 2007; Chavarria et al., 2014). In eukaryotes, Ncs6 forms a heterocomplex with the Ncs2 protein that appears to have a role beyond catalysis (Esberg et al., 2006; Dewez et al., 2008). In some thermophilic bacteria and archaea, such as *T. thermophilus*, *Thermotoga maritima*, and *Pyrococcus horikoshii*, TtuA catalyzes the same 2-thiouridylation reaction at different positions (e.g., position 54) (Shigi et al., 2006a; Arragain et al., 2017). Although Ncs6/TtuA has a PP-loop motif and requires ATP for activity, the sulfur transfer mechanism (Figure 2C) is markedly different from that of MnmA in two aspects: Ncs6/TtuA utilizes an oxygen-sensitive Fe-S cluster and a unique thiocarboxylate (R-COSH) that is formed on the carboxy terminus of the sulfur carrier protein Urm1/TtuB, believed to be ancient ubiquitin-like post-translational modifiers (Shigi et al., 2008; Leidel et al., 2009; Shigi, 2012). The C-terminus of Urm1/TtuB is thiocarboxylated with a sulfur atom from free L-cysteine via an adenylated intermediate catalyzed by the E1-like enzyme Uba4/TtuC. Meanwhile, Tum1/TtuD enhances the activity of cysteine desulfurases and directs sulfur flow to s^2^U biosynthesis (Shigi et al., 2006b; Noma et al., 2009; Shigi et al., 2016).

In TtuA, a [4Fe-4S] cluster is ligated by three conserved cysteines, leaving one iron atom free for ligand binding, which may be important in the sulfur transfer reaction (Figure 2D; Nakagawa et al., 2013; Arragain et al., 2017; Chen et al., 2017). TtuA activates the C2 position of U54 by forming an acyl-adenylated intermediate. The thiocarboxylate of TtuB is subsequently attached near the adenylate by the iron-sulfur cluster, and the sulfur atom then attacks the C2 position of the uridine (Figure 2C (1)), forming the s^2^U product. In an alternative mechanism, a sulfide ion released from TtuB-COSH may bind to the free iron atom of the Fe-S cluster, and become incorporated into s^2^U (Figure 2C (2)). The latter pathway may be utilized by organisms lacking a TtuB homolog, and the sulfide could be derived from free sulfide ions in the cell. In support of this mechanism, sulfur atoms from free sulfides were incorporated *in vitro*, and a sulfide captured by the iron-sulfur cluster was observed in the crystal structure (Arragain et al., 2017), although direct proof *in vivo* has not yet been obtained. Changes in the electronic properties of the Fe-S cluster during the reaction should also be investigated to understand the role in sulfur transfer.

It has also been demonstrated that *Saccharomyces cerevisiae* Ncs6 and *Methanococcus maripaludis* NcsA can bind [3Fe-4S] clusters (Liu et al., 2016). TtcA, which has a 2-thiocytidylase activity at position 32 and belongs to a subgroup of the Ncs6/TtuA family, also requires the [4Fe-4S] cluster for catalysis (Jager et al., 2004; Bouvier et al., 2014). In addition, *M. maripaludis* ThiI has a [3Fe-4S] cluster that is essential for catalysis (Liu et al., 2016). Knowledge of the functional differences and distributions of [4Fe-4S], [3Fe-4S], and other cluster types in these enzymes may lead to a better understanding of the precise mechanisms of Fe-S cluster-dependent sulfurtransferases. Although it was revealed that TtuA recognizes a common T-loop sequence in tRNAs (Shigi et al., 2002), the structural basis of tRNA recognition by Ncs6/TtuA family enzymes remains to be elucidated.

## The Role of Radical *S*-Adenosylmethionine (Rsam) Enzyme Miab in 2-Methylthio a Synthesis

Methylthio-A37 methylthiotransferases such as MiaB in bacteria and its paralogs in eukaryotes (Esberg et al., 1999; Pierrel et al., 2002; Arragain et al., 2010) are a subgroup of rSAM enzymes that possesses two Fe-S clusters (Lanz and Booker, 2015). rSAM enzymes catalyze the reductive cleavage of SAM to methionine and the highly reactive 5′-deoxyadenosyl (5′-dA) radical using a [4Fe-4S] cluster, called the “rSAM cluster.” By abstracting a hydrogen atom from the substrate, the 5′-dA radical generates a substrate radical intermediate (Figure 2E). The rSAM cluster and an additional auxiliary [4Fe-4S] cluster (the “Aux cluster”) are ligated by two sets of three conserved cysteine residues, and located near each other; the distance between the two clusters is ∼8 Å in the structurally characterized related enzyme RimO (Forouhar et al., 2013; Figure 2F), which catalyzes the insertion of a methylthio group on the Asp89 residue of the bacterial ribosomal protein S12 (Anton et al., 2008). Interestingly, the two ligand-free iron atoms of the rSAM and Aux clusters are bridged by a pentasulfide chain in this structure. It was proposed that this bridging sulfur mimics the sulfur donor, and the sulfur does not appear to come from the iron-sulfur clusters themselves, but the exact nature of the sulfur donor remains to be determined. An explanation of the reaction mechanism has been proposed in which the transfer of a methyl group from another molecule of SAM to the sulfur atom of the tip of the polysulfide attached to the Aux cluster is followed by attack of a substrate radical on the methylated sulfur atom to generate ms^2^A (Landgraf et al., 2013; Figure 2E). Recently, a hypermodified nucleoside, 2-methylthiomethylenethio-A (msms^2^A), was identified in *E. coli* tRNAs, and MiaB is involved in msms^2^A synthesis (Dal Magro et al., 2018). MiaB may abstract a hydrogen radical from the methyl group of ms^2^A, which is introduced in the first step of the reaction, and a second methylthio transfer reaction could then follow.

## Relationships With Other Sulfur-Containing Metabolites

The use of protein-thiocarboxylate intermediates (MoaD-COSH and ThiS-COSH) in the biosynthesis of sulfur-containing essential metabolites such as Moco and thiamin was revealed by pioneering research by the groups of Rajagopalan (Leimkuhler et al., 2011) and Begley (Settembre et al., 2003), respectively. As described above, tRNA sulfurtransferase Ncs6/TtuA also utilizes Urm1/TtuB-COSH as a sulfur donor. In some bacteria, thiocarboxylates are utilized, as demonstrated in the biosynthesis of L-cysteine in *Mycobacterium tuberculosis* (Burns et al., 2005) and L-methionine in *Wolinella succinogenes* (Krishnamoorthy and Begley, 2011).

In addition to these primary metabolites, there are numerous other C-S bond-containing natural products (Figure 1C), the biosynthesis of which has been comprehensively reviewed (Dunbar et al., 2017). Protein thiocarboxylates are utilized in the biosynthesis of siderophores in some *Pseudomonas* (Matthijs et al., 2004), such as pyridine-2,6-dithiocarboxylic acid and thioquinolobactin, and the antibiotic BE-7585A in *Amycolatopsis orientalis*, which contains a 2-thiosugar moiety (Sasaki et al., 2014). Although the biosynthetic gene clusters for these siderophores contain a pathway-specific ThiS paralog, the BE-7585A biosynthetic gene cluster does not. Instead, the 2-thiosugar is synthesized by borrowing sulfur carrier proteins from L-cysteine and Moco biosynthesis.

Interestingly, in the biosynthesis of the thiotetronate antibiotics such as thiolactomycin and Tü 3010 (Tao et al., 2016), the backbone polyketide is synthesized by a polyketide synthase (PKS) and a nonribosomal peptide synthase (NRPS) encoded in the biosynthetic operon. Remarkably, *in vivo* experiments showed that the sulfur atom in Tü 3010 may be incorporated by a cysteine desulfurase and MnmA, separately encoded from the biosynthetic operon, which are also probably involved in s^2^U biosynthesis in tRNAs. The involvement of these genes in s^2^U synthesis should be experimentally validated, it would therefore be interesting to decipher the mechanism by which MnmA specifically recognizes and incorporates the sulfur atom in the precursor of thiotetronate, in addition to its cognate tRNA substrates. Alternatively, an additional sulfur carrier protein(s) may mediate between the MnmA and thiotetronate biosynthesis machinery. The 6-thioguanosine (s^6^G) modification is a virulence factor in the plant pathogen *Erwinia amylovorans*, and two proteins are required for the formation of s^6^G both *in vivo* and *in vitro* (Litomska et al., 2018). The first, YcfC, is distantly related to PLP-dependent transferases such as cysteine desulfurases and carbon-sulfur lyases. The second, YcfA, is a PP-loop-containing ATPase distantly related to, and perhaps evolved from, tRNA modification enzymes such as MnmA, ThiI, Ncs6/TtuA, and TtcA. Furthermore, YcaO forms a thiolate phosphorylated intermediate similar to that formed by the PP-loop ATPase, representing another interesting example of the biosynthesis of thioamide-containing natural products (Mahanta et al., 2018).

## Perspectives

This review summarizes recent advances in our understanding of the sulfur-related modification of RNA. Recent studies reveal the widespread involvement of modification enzymes with Fe-S clusters in all three domains of life. Because Fe-S clusters and sulfur modifications themselves (Sierant et al., 2018a) are sensitive to cellular oxidative stress, sulfur modifications may carefully be regulated by cellular oxidative status. Differences in the stability of protein persulfides and protein thiocarboxylates in cells may be important and require investigation in the future. Cellular sulfur donors mediating sulfur-related modification of tRNAs are believed to be derived from free L-cysteine (Lauhon and Kambampati, 2000; Lauhon, 2002; Nilsson et al., 2002; Shigi et al., 2006b), and numerous types of cellular-free persulfides such as L-Cys-SSH have been discovered (Ida et al., 2014; Akaike et al., 2017). The ms^2^A modification is regulated by L-Cys-SSH in mammalian cells (Takahashi et al., 2017), while free sulfide is proposed to serve as a sulfur donor in archaea (Liu et al., 2010). Because sulfur atoms can adopt diverse chemical forms and partake in a wide range of reactions, it is important to exercise great caution when attempting to identify the actual *in vivo* sulfur donors responsible for the biosynthesis of sulfur-containing biomolecules.

The roles of thiocarboxylate sulfur-carrier proteins have been characterized in the biosynthesis of primary metabolites, leading to the discovery of their roles in those of secondary metabolites. Regarding sulfur carriers and/or other components shared by several biosynthetic pathway, the regulation mechanism of sulfur-flow to each pathway may be interesting and worthy of exploration, especially between primary and secondary metabolites. Strategy utilizing carrier proteins are not limited to the biosynthesis pathway of sulfur-containing molecules, it is more general strategies in life. In L-lysine synthesis in *Thermus thermophilus* (Horie et al., 2009) and L-lysine/L-arginine synthesis in *Sulfolobus acidocaldarius* (Ouchi et al., 2013), “amino-group carrier proteins (AmCPs)” are utilized for carrying reaction intermediates, which prevents unwanted intramolecular reactions and enables successive reaction steps to proceed efficiently. Recently, AmCPs have also been identified as parts of the machinery producing the natural product diamino-dihydroxy-heptanoic acid in *Streptomyces* species (Matsuda et al., 2017).

The biosynthetic pathways underpinning sulfur modification of RNA in all domains of life share many aspects in common; hence research on bacteria can strengthen our understanding of this process in eukaryotes, including humans. Dysfunctional RNA modification, especially involving anticodons, can lead to diseases (Shigi, 2016). Abnormalities in RNA modification are caused by three main factors: (1) mutations in genes encoding modification enzymes, (2) mutations in substrate tRNAs, and (3) alterations in metabolites acting as substrates. The cytosolic ms^2^A modification is required for the production of proinsulin, which explains why single-nucleotide polymorphisms (SNPs) in the *Cdkal1* gene (a *MiaB* homolog) are a risk factor for type II diabetes (Wei et al., 2011). Mutation of the *Mtu1* gene causes abnormalities in s^2^U modifications, and leads to the mitochondrial disease reversible infantile liver failure (RILF) (Wu et al., 2016). Similarly, in the mitochondrial disease myoclonic epilepsy with red ragged fibers (MERRF), a point mutation in mt-tRNA-Lys leads to the abnormal modification of its anticodon, resulting in disease (Kirino and Suzuki, 2005).

## Author Contributions

NS designed the study and wrote the manuscript.

## Conflict of Interest Statement

The author declares that the research was conducted in the absence of any commercial or financial relationships that could be construed as a potential conflict of interest.

## References

[B1] Agris P. F. Sierzputowska-Gracz H. Smith W. Malkiewicz A. Sochacka E. Nawrot B. (1992). Thiolation of uridine carbon-2 restricts the motional dynamics of the transfer RNA wobble position nucleoside. *J. Am. Chem. Soc.* 114 2652–2656. 10.1021/ja00033a044

[B2] Agris P. F. Soll D. Seno T. (1973). Biological function of 2-thiouridine in *Escherichia coli* glutamic acid transfer ribonucleic acid. *Biochemistry* 12 4331–4337. 10.1021/bi00746a005 4584321

[B3] Akaike T. Ida T. Wei F. Y. Nishida M. Kumagai Y. Alam M. M. (2017). Cysteinyl-tRNA synthetase governs cysteine polysulfidation and mitochondrial bioenergetics. *Nat. Commun.* 8:1177. 10.1038/s41467-017-01311-y 29079736 PMC5660078

[B4] Anton B. P. Saleh L. Benner J. S. Raleigh E. A. Kasif S. Roberts R. J. (2008). RimO, a MiaB-like enzyme, methylthiolates the universally conserved Asp88 residue of ribosomal protein S12 in *Escherichia coli*. *Proc. Natl. Acad. Sci. U.S.A.* 105 1826–1831. 10.1073/pnas.0708608105 18252828 PMC2538847

[B5] Arragain S. Bimai O. Legrand P. Caillat S. Ravanat J. L. Touati N. (2017). Nonredox thiolation in tRNA occurring via sulfur activation by a [4Fe-4S] cluster. *Proc. Natl. Acad. Sci. U.S.A.* 114 7355–7360. 10.1073/pnas.1700902114 28655838 PMC5514717

[B6] Arragain S. Handelman S. K. Forouhar F. Wei F. Y. Tomizawa K. Hunt J. F. (2010). Identification of eukaryotic and prokaryotic methylthiotransferase for biosynthesis of 2-methylthio-N6-threonylcarbamoyladenosine in tRNA. *J. Biol. Chem.* 285 28425–28433. 10.1074/jbc.M110.106831 20584901 PMC2937867

[B7] Aucynaite A. Rutkiene R. Gasparaviciute R. Meskys R. Urbonavicius J. (2018). A gene encoding a DUF523 domain protein is involved in the conversion of 2-thiouracil into uracil. *Environ. Microbiol. Rep.* 10 49–56. 10.1111/1758-2229.12605 29194984

[B8] Bjork G. R. Huang B. Persson O. P. Bystrom A. S. (2007). A conserved modified wobble nucleoside (mcm5s2U) in lysyl-tRNA is required for viability in yeast. *RNA* 13 1245–1255. 10.1261/rna.558707 17592039 PMC1924908

[B9] Black K. A. Dos Santos P. C. (2015). Abbreviated pathway for biosynthesis of 2-thiouridine in *Bacillus subtilis*. *J. Bacteriol.* 197 1952–1962. 10.1128/JB.02625-14 25825430 PMC4420905

[B10] Boccaletto P. Machnicka M. A. Purta E. Piatkowski P. Baginski B. Wirecki T. K. (2018). MODOMICS: a database of RNA modification pathways. 2017 update. *Nucleic Acids Res.* 46 D303–D307. 10.1093/nar/gkx1030 29106616 PMC5753262

[B11] Bouvier D. Labessan N. Clemancey M. Latour J. M. Ravanat J. L. Fontecave M. (2014). TtcA a new tRNA-thioltransferase with an Fe-S cluster. *Nucleic Acids Res.* 42 7960–7970. 10.1093/nar/gku508 24914049 PMC4081106

[B12] Burns K. E. Baumgart S. Dorrestein P. C. Zhai H. McLafferty F. W. Begley T. P. (2005). Reconstitution of a new cysteine biosynthetic pathway in *Mycobacterium tuberculosis*. *J. Am. Chem. Soc.* 127 11602–11603. 10.1021/ja053476x 16104727 PMC2536522

[B13] Burrows W. J. Armstrong D. J. Skoog F. Hecht S. M. Boyle J. T. Leonard N. J. (1968). Cytokinin from soluble RNA of *Escherichia coli*: 6-(3-methyl-2-butenylamino)-2-methylthio-9-beta-D-ribofuranosylpurine. *Science* 161 691–693. 10.1126/science.161.3842.691 4874577

[B14] Cantara W. A. Crain P. F. Rozenski J. McCloskey J. A. Harris K. A. Zhang X. (2011). The RNA Modification Database, RNAMDB: 2011 update. *Nucleic Acids Res.* 39 D195–D201. 10.1093/nar/gkq1028 21071406 PMC3013656

[B15] Carbon J. David H. Studier M. H. (1968). Thiobases in *Escherchia coli* Transfer RNA: 2-Thiocytosine and 5-Methylaminomethyl-2-thiouracil. *Science* 161 1146–1147. 10.1126/science.161.3846.1146 17812290

[B16] Carre D. S. Thomas G. Favre A. (1974). Conformation and functioning of tRNAs: cross-linked tRNAs as substrate for tRNA nucleotidyl-transferase and aminoacyl synthetases. *Biochimie* 56 1089–1101. 10.1016/S0300-9084(74)80097-0 4614866

[B17] Chavarria N. E. Hwang S. Cao S. Fu X. Holman M. Elbanna D. (2014). Archaeal Tuc1/Ncs6 homolog required for wobble uridine tRNA thiolation is associated with ubiquitin-proteasome, translation, and RNA processing system homologs. *PLoS One* 9:e99104. 10.1371/journal.pone.0099104 24906001 PMC4048286

[B18] Chen M. Asai S. I. Narai S. Nambu S. Omura N. Sakaguchi Y. (2017). Biochemical and structural characterization of oxygen-sensitive 2-thiouridine synthesis catalyzed by an iron-sulfur protein TtuA. *Proc. Natl. Acad. Sci. U.S.A.* 114 4954–4959. 10.1073/pnas.1615585114 28439027 PMC5441745

[B19] Chen P. Crain P. F. Nasvall S. J. Pomerantz S. C. Bjork G. R. (2005). A “gain of function” mutation in a protein mediates production of novel modified nucleosides. *EMBO J.* 24 1842–1851. 10.1038/sj.emboj.7600666 15861125 PMC1142597

[B20] Chionh Y. H. McBee M. Babu I. R. Hia F. Lin W. Zhao W. (2016). tRNA-mediated codon-biased translation in mycobacterial hypoxic persistence. *Nat. Commun.* 7:13302. 10.1038/ncomms13302 27834374 PMC5114619

[B21] Crain P. F. Alfonzo J. D. Rozenski J. Kapushoc S. T. McCloskey J. A. Simpson L. (2002). Modification of the universally unmodified uridine-33 in a mitochondria-imported edited tRNA and the role of the anticodon arm structure on editing efficiency. *RNA* 8 752–761. 10.1017/S1355838202022045 12088148 PMC1370294

[B22] Dahl J. U. Radon C. Buhning M. Nimtz M. Leichert L. I. Denis Y. (2013). The sulfur carrier protein TusA has a pleiotropic role in *Escherichia coli* that also affects molybdenum cofactor biosynthesis. *J. Biol. Chem.* 288 5426–5442. 10.1074/jbc.M112.431569 23281480 PMC3581435

[B23] Dal Magro C. Keller P. Kotter A. Werner S. Duarte V. Marchand V. (2018). A vastly increased chemical variety of RNA modifications containing a thioacetal structure. *Angew. Chem. Int. Ed. Engl.* 57 7893–7897. 10.1002/anie.201713188 29624844

[B24] Dewez M. Bauer F. Dieu M. Raes M. Vandenhaute J. Hermand D. (2008). The conserved Wobble uridine tRNA thiolase Ctu1-Ctu2 is required to maintain genome integrity. *Proc. Natl. Acad. Sci. U.S.A.* 105 5459–5464. 10.1073/pnas.0709404105 18391219 PMC2291126

[B25] Dumelin C. E. Chen Y. Leconte A. M. Chen Y. G. Liu D. R. (2012). Discovery and biological characterization of geranylated RNA in bacteria. *Nat. Chem. Biol.* 8 913–919. 10.1038/nchembio.1070 22983156 PMC3494293

[B26] Dunbar K. L. Scharf D. H. Litomska A. Hertweck C. (2017). Enzymatic carbon-sulfur bond formation in natural product biosynthesis. *Chem. Rev.* 117 5521–5577. 10.1021/acs.chemrev.6b00697 28418240

[B27] Durant P. C. Bajji A. C. Sundaram M. Kumar R. K. Davis D. R. (2005). Structural effects of hypermodified nucleosides in the *Escherichia coli* and human tRNALys anticodon loop: the effect of nucleosides s2U, mcm5U, mcm5s2U, mnm5s2U, t6A, and ms2t6A. *Biochemistry* 44 8078–8089. 10.1021/bi050343f 15924427

[B28] Elseviers D. Petrullo L. A. Gallagher P. J. (1984). Novel *E. coli* mutants deficient in biosynthesis of 5-methylaminomethyl-2-thiouridine. *Nucleic Acids Res.* 12 3521–3534. 10.1093/nar/12.8.3521 6427754 PMC318766

[B29] Esberg A. Huang B. Johansson M. J. Bystrom A. S. (2006). Elevated levels of two tRNA species bypass the requirement for elongator complex in transcription and exocytosis. *Mol. Cell.* 24 139–148. 10.1016/j.molcel.2006.07.031 17018299

[B30] Esberg B. Leung H. C. Tsui H. C. Bjork G. R. Winkler M. E. (1999). Identification of the miaB gene, involved in methylthiolation of isopentenylated A37 derivatives in the tRNA of *Salmonella typhimurium* and *Escherichia coli*. *J. Bacteriol.* 181 7256–7265. 10572129 10.1128/jb.181.23.7256-7265.1999 PMC103688

[B31] Favre A. Yaniv M. Michelson A. M. (1969). The photochemistry of 4-thiouridine in *Escherichia coli* t-RNA Val1. *Biochem. Biophys. Res. Commun.* 37 266–271. 10.1016/0006-291X(69)90729-3 4898696

[B32] Flint D. H. (1996). *Escherichia coli* contains a protein that is homologous in function and N-terminal sequence to the protein encoded by the nifS gene of *Azotobacter vinelandii* and that can participate in the synthesis of the Fe-S cluster of dihydroxy-acid dehydratase. *J. Biol. Chem.* 271 16068–16074. 8663056

[B33] Forouhar F. Arragain S. Atta M. Gambarelli S. Mouesca J. M. Hussain M. (2013). Two Fe-S clusters catalyze sulfur insertion by radical-SAM methylthiotransferases. *Nat. Chem. Biol.* 9 333–338. 10.1038/nchembio.1229 23542644 PMC4118475

[B34] Griffey R. H. Davis D. R. Yamaizumi Z. Nishimura S. Hawkins B. L. Poulter C. D. (1986). 15N-labeled tRNA. Identification of 4-thiouridine in *Escherichia coli* tRNASer1 and tRNATyr2 by 1H-15N two-dimensional NMR spectroscopy. *J. Biol. Chem.* 261 12074–12078. 3638307

[B35] Hidese R. Mihara H. Esaki N. (2011). Bacterial cysteine desulfurases: versatile key players in biosynthetic pathways of sulfur-containing biofactors. *Appl. Microbiol. Biotechnol.* 91 47–61. 10.1007/s00253-011-3336-x 21603932

[B36] Horie A. Tomita T. Saiki A. Kono H. Taka H. Mineki R. (2009). Discovery of proteinaceous N-modification in lysine biosynthesis of *Thermus thermophilus*. *Nat. Chem. Biol.* 5 673–679. 10.1038/nchembio.198 19620981

[B37] Horie N. Hara-Yokoyama M. Yokoyama S. Watanabe K. Kuchino Y. Nishimura S. (1985). Two tRNAIle1 species from an extreme thermophile, *Thermus thermophilus* HB8: effect of 2-thiolation of ribothymidine on the thermostability of tRNA. *Biochemistry* 24 5711–5715. 10.1021/bi00342a004 3853464

[B38] Ida T. Sawa T. Ihara H. Tsuchiya Y. Watanabe Y. Kumagai Y. (2014). Reactive cysteine persulfides and S-polythiolation regulate oxidative stress and redox signaling. *Proc. Natl. Acad. Sci. U.S.A.* 111 7606–7611. 10.1073/pnas.1321232111 24733942 PMC4040604

[B39] Ikeuchi Y. Shigi N. Kato J. Nishimura A. Suzuki T. (2006). Mechanistic insights into sulfur relay by multiple sulfur mediators involved in thiouridine biosynthesis at tRNA wobble positions. *Mol. Cell.* 21 97–108. 10.1016/j.molcel.2005.11.001 16387657

[B40] Ishikura H. Yamada Y. Nishimura S. (1971). The nucleotide sequence of a serine tRNA from *Escherichia coli*. *FEBS Lett.* 16 68–70. 10.1016/0014-5793(71)80688-9 11945903

[B41] Jager G. Leipuviene R. Pollard M. G. Qian Q. Bjork G. R. (2004). The conserved Cys-X1-X2-Cys motif present in the TtcA protein is required for the thiolation of cytidine in position 32 of tRNA from *Salmonella enterica Serovar typhimurium*. *J. Bacteriol.* 186 750–757. 10.1128/JB.186.3.750-757.2004 14729701 PMC321475

[B42] Jenner L. B. Demeshkina N. Yusupova G. Yusupov M. (2010). Structural aspects of messenger RNA reading frame maintenance by the ribosome. *Nat. Struct. Mol. Biol.* 17 555–560. 10.1038/nsmb.1790 20400952

[B43] Johansson M. J. Esberg A. Huang B. Bjork G. R. Bystrom A. S. (2008). Eukaryotic wobble uridine modifications promote a functionally redundant decoding system. *Mol. Cell. Biol.* 28 3301–3312. 10.1128/MCB.01542-07 18332122 PMC2423140

[B44] Kambampati R. Lauhon C. T. (2003). MnmA and IscS are required for in vitro 2-thiouridine biosynthesis in *Escherichia coli*. *Biochemistry* 42 1109–1117. 10.1021/bi026536+ 12549933

[B45] Kirino Y. Suzuki T. (2005). Human mitochondrial diseases associated with tRNA wobble modification deficiency. *RNA Biol.* 2 41–44. 10.4161/rna.2.2.1610 17132941

[B46] Kowalak J. A. Dalluge J. J. McCloskey J. A. Stetter K. O. (1994). The role of posttranscriptional modification in stabilization of transfer RNA from hyperthermophiles. *Biochemistry* 33 7869–7876. 10.1021/bi00191a014 7516708

[B47] Krishnamoorthy K. Begley T. P. (2011). Protein thiocarboxylate-dependent methionine biosynthesis in *Wolinella succinogenes*. *J. Am. Chem. Soc.* 133 379–386. 10.1021/ja107424t 21162571 PMC3089676

[B48] Landgraf B. J. Arcinas A. J. Lee K. H. Booker S. J. (2013). Identification of an intermediate methyl carrier in the radical S-adenosylmethionine methylthiotransferases RimO and MiaB. *J. Am. Chem. Soc.* 135 15404–15416. 10.1021/ja4048448 23991893 PMC4023531

[B49] Lanz N. D. Booker S. J. (2015). Auxiliary iron-sulfur cofactors in radical SAM enzymes. *Biochim. Biophys. Acta* 1853 1316–1334. 10.1016/j.bbamcr.2015.01.002 25597998

[B50] Lauhon C. T. (2002). Requirement for IscS in biosynthesis of all thionucleosides in *Escherichia coli*. *J. Bacteriol.* 184 6820–6829. 10.1128/JB.184.24.6820-6829.2002 12446632 PMC135461

[B51] Lauhon C. T. Kambampati R. (2000). The iscS gene in *Escherichia coli* is required for the biosynthesis of 4-thiouridine, thiamin, and NAD. *J. Biol. Chem.* 275 20096–20103. 10.1074/jbc.M002680200 10781607

[B52] Lauhon C. T. Skovran E. Urbina H. D. Downs D. M. Vickery L. E. (2004). Substitutions in an active site loop of *Escherichia coli* IscS result in specific defects in Fe-S cluster and thionucleoside biosynthesis in vivo. *J. Biol. Chem.* 279 19551–19558. 10.1074/jbc.M401261200 14978044

[B53] Laxman S. Sutter B. M. Wu X. Kumar S. Guo X. Trudgian D. C. (2013). Sulfur amino acids regulate translational capacity and metabolic homeostasis through modulation of tRNA thiolation. *Cell* 154 416–429. 10.1016/j.cell.2013.06.043 23870129 PMC3757545

[B54] Leidel S. Pedrioli P. G. Bucher T. Brost R. Costanzo M. Schmidt A. (2009). Ubiquitin-related modifier Urm1 acts as a sulphur carrier in thiolation of eukaryotic transfer RNA. *Nature* 458 228–232. 10.1038/nature07643 19145231

[B55] Leimkuhler S. Wuebbens M. M. Rajagopalan K. V. (2011). The history of the discovery of the molybdenum cofactor and novel aspects of its biosynthesis in bacteria. *Coord. Chem. Rev.* 255 1129–1144. 10.1016/j.ccr.2010.12.003 21528011 PMC3081585

[B56] Leipuviene R. Qian Q. Bjork G. R. (2004). Formation of thiolated nucleosides present in tRNA from *Salmonella enterica Serovar typhimurium* occurs in two principally distinct pathways. *J. Bacteriol.* 186 758–766. 10.1128/JB.186.3.758-766.2004 14729702 PMC321476

[B57] Lipsett M. N. (1965). The isolation of 4-thiouridylic acid from the soluble ribonucleic acid of *Escherichia coli*. *J. Biol. Chem.* 240 3975–3978. 5320644

[B58] Litomska A. Ishida K. Dunbar K. L. Boettger M. Coyne S. Hertweck C. (2018). Enzymatic thioamide formation in bacterial antimetabolite pathway. *Angew Chem. Int. Ed. Engl.* 57 36. 10.1002/anie.201804158 29947149

[B59] Liu Y. Sieprawska-Lupa M. Whitman W. B. White R. H. (2010). Cysteine is not the sulfur source for iron-sulfur cluster and methionine biosynthesis in the methanogenic archaeon *Methanococcus maripaludis*. *J. Biol. Chem.* 285 31923–31929. 10.1074/jbc.M110.152447 20709756 PMC2952193

[B60] Liu Y. Vinyard D. J. Reesbeck M. E. Suzuki T. Manakongtreecheep K. Holland P. L. (2016). A [3Fe-4S] cluster is required for tRNA thiolation in archaea and eukaryotes. *Proc. Natl. Acad. Sci. U.S.A.* 113 12703–12708. 10.1073/pnas.1615732113 27791189 PMC5111681

[B61] Liu Y. Zhu X. Nakamura A. Orlando R. Soll D. Whitman W. B. (2012). Biosynthesis of 4-thiouridine in tRNA in the methanogenic archaeon *Methanococcus maripaludis*. *J. Biol. Chem.* 287 36683–36692. 10.1074/jbc.M112.405688 22904325 PMC3481272

[B62] Mahanta N. Liu A. Dong S. Nair S. K. Mitchell D. A. (2018). Enzymatic reconstitution of ribosomal peptide backbone thioamidation. *Proc. Natl. Acad. Sci. U.S.A.* 115 3030–3035. 10.1073/pnas.1722324115 29507203 PMC5866606

[B63] Matsuda K. Hasebe F. Shiwa Y. Kanesaki Y. Tomita T. Yoshikawa H. (2017). Genome mining of amino group carrier protein-mediated machinery: discovery and biosynthetic characterization of a natural product with unique hydrazone unit. *ACS Chem. Biol.* 12 124–131. 10.1021/acschembio.6b00818 28103675

[B64] Matthijs S. Baysse C. Koedam N. Tehrani K. A. Verheyden L. Budzikiewicz H. (2004). The Pseudomonas siderophore quinolobactin is synthesized from xanthurenic acid, an intermediate of the kynurenine pathway. *Mol. Microbiol.* 52 371–384. 10.1111/j.1365-2958.2004.03999.x 15066027

[B65] Maynard N. D. Macklin D. N. Kirkegaard K. Covert M. W. (2012). Competing pathways control host resistance to virus via tRNA modification and programmed ribosomal frameshifting. *Mol. Syst. Biol.* 8:567. 10.1038/msb.2011.101 22294093 PMC3296357

[B66] Mueller E. G. (2006). Trafficking in persulfides: delivering sulfur in biosynthetic pathways. *Nat. Chem. Biol.* 2 185–194. 10.1038/nchembio779 16547481

[B67] Mueller E. G. Buck C. J. Palenchar P. M. Barnhart L. E. Paulson J. L. (1998). Identification of a gene involved in the generation of 4-thiouridine in tRNA. *Nucleic Acids Res.* 26 2606–2610. 10.1093/nar/26.11.2606 9592144 PMC147624

[B68] Mueller E. G. Palenchar P. M. (1999). Using genomic information to investigate the function of ThiI, an enzyme shared between thiamin and 4-thiouridine biosynthesis. *Protein Sci.* 8 2424–2427. 10.1110/ps.8.11.2424 10595545 PMC2144177

[B69] Murao K. Tanabe T. Ishii F. Namiki M. Nishimura S. (1972). Primary sequence of arginine transfer RNA from *Escherichia coli*. *Biochem. Biophys. Res. Commun.* 47 1332–1337. 10.1016/0006-291X(72)90218-5 4557171

[B70] Murphy F. V. Ramakrishnan V. Malkiewicz A. Agris P. F. (2004). The role of modifications in codon discrimination by tRNA(Lys)UUU. *Nat. Struct. Mol. Biol.* 11 1186–1191. 10.1038/nsmb861 15558052

[B71] Nakagawa H. Kuratani M. Goto-Ito S. Ito T. Katsura K. Terada T. (2013). Crystallographic and mutational studies on the tRNA thiouridine synthetase TtuA. *Proteins* 81 1232–1244. 10.1002/prot.24273 23444054

[B72] Nakai Y. Nakai M. Lill R. Suzuki T. Hayashi H. (2007). Thio modification of yeast cytosolic tRNA is an iron-sulfur protein-dependent pathway. *Mol. Cell. Biol.* 27 2841–2847. 10.1128/MCB.01321-06 17283054 PMC1899921

[B73] Nedialkova D. D. Leidel S. A. (2015). Optimization of codon translation rates via tRNA modifications maintains proteome integrity. *Cell* 161 1606–1618. 10.1016/j.cell.2015.05.022 26052047 PMC4503807

[B74] Neumann P. Lakomek K. Naumann P. T. Erwin W. M. Lauhon C. T. Ficner R. (2014). Crystal structure of a 4-thiouridine synthetase-RNA complex reveals specificity of tRNA U8 modification. *Nucleic Acids Res.* 42 6673–6685. 10.1093/nar/gku249 24705700 PMC4041423

[B75] Nilsson K. Lundgren H. K. Hagervall T. G. Bjork G. R. (2002). The cysteine desulfurase IscS is required for synthesis of all five thiolated nucleosides present in tRNA from *Salmonella enterica Serovar typhimurium*. *J. Bacteriol.* 184 6830–6835. 10.1128/JB.184.24.6830-6835.2002 12446633 PMC135462

[B76] Noma A. Sakaguchi Y. Suzuki T. (2009). Mechanistic characterization of the sulfur-relay system for eukaryotic 2-thiouridine biogenesis at tRNA wobble positions. *Nucleic Acids Res.* 37 1335–1352. 10.1093/nar/gkn1023 19151091 PMC2651780

[B77] Numata T. Ikeuchi Y. Fukai S. Suzuki T. Nureki O. (2006). Snapshots of tRNA sulphuration via an adenylated intermediate. *Nature* 442 419–424. 10.1038/nature04896 16871210

[B78] Oashi Z. Saneyoshi M. Harada F. Hara H. Nishimura S. (1970). Presumed anticodon structure of glutamic acid tRNA from E. coli: a possible location of a 2-thiouridine derivative in the first position of the anticodon. *Biochem. Biophys. Res. Commun.* 40 866–872. 10.1016/0006-291X(70)90983-6 4924671

[B79] Ouchi T. Tomita T. Horie A. Yoshida A. Takahashi K. Nishida H. (2013). Lysine and arginine biosyntheses mediated by a common carrier protein in Sulfolobus. *Nat. Chem. Biol.* 9 277–283. 10.1038/nchembio.1200 23434852

[B80] Pierrel F. Bjork G. R. Fontecave M. Atta M. (2002). Enzymatic modification of tRNAs: MiaB is an iron-sulfur protein. *J. Biol. Chem.* 277 13367–13370. 10.1074/jbc.C100609200 11882645

[B81] Rajakovich L. J. Tomlinson J. Dos Santos P. C. (2012). Functional analysis of *Bacillus subtilis* genes involved in the biosynthesis of 4-Thiouridine in tRNA. *J. Bacteriol.* 194 4933–4940. 10.1128/JB.00842-12 22773787 PMC3430334

[B82] Ryals J. Hsu R. Y. Lipsett M. N. Bremer H. (1982). Isolation of single-site *Escherichia coli* mutants deficient in thiamine and 4-thiouridine syntheses: identification of a nuvC mutant. *J. Bacteriol.* 151 899–904. 6178725 10.1128/jb.151.2.899-904.1982 PMC220340

[B83] Sasaki E. Zhang X. Sun H. G. Lu M. Y. Liu T. L. Ou A. (2014). Co-opting sulphur-carrier proteins from primary metabolic pathways for 2-thiosugar biosynthesis. *Nature* 510 427–431. 10.1038/nature13256 24814342 PMC4082789

[B84] Schindelin H. Kisker C. Rajagopalan K. V. (2001). Molybdopterin from molybdenum and tungsten enzymes. *Adv. Protein Chem.* 58 47–94. 10.1016/S0065-3233(01)58002-X 11665493

[B85] Settembre E. Begley T. P. Ealick S. E. (2003). Structural biology of enzymes of the thiamin biosynthesis pathway. *Curr. Opin. Struct. Biol.* 13 739–747. 10.1016/j.sbi.2003.10.006 14675553

[B86] Shi R. Proteau A. Villarroya M. Moukadiri I. Zhang L. Trempe J. F. (2010). Structural basis for Fe-S cluster assembly and tRNA thiolation mediated by IscS protein-protein interactions. *PLoS Biol.* 8:e1000354. 10.1371/journal.pbio.1000354 20404999 PMC2854127

[B87] Shigi N. (2012). Posttranslational modification of cellular proteins by a ubiquitin-like protein in bacteria. *J. Biol. Chem.* 287 17568–17577. 10.1074/jbc.M112.359844 22467871 PMC3366818

[B88] Shigi N. (2014). Biosynthesis and functions of sulfur modifications in tRNA. *Front Genet* 5:67 10.3389/fgene.2014.00067 PMC3980101 24765101

[B89] Shigi N. (2016). “Sulfur modifications in tRNA: function and implications for human disease,” in *Modified Nucleic Acids in Biology and Medicine*, eds Jurga S. Erdmann V. A. Barciszewski J. (Berlin: Springer), 55–71. 10.1007/978-3-319-34175-0_3

[B90] Shigi N. Asai S. I. Watanabe K. (2016). Identification of a rhodanese-like protein involved in thiouridine biosynthesis in *Thermus thermophilus* tRNA. *FEBS Lett.* 590 4628–4637. 10.1002/1873-3468.12499 27878988

[B91] Shigi N. Sakaguchi Y. Asai S. Suzuki T. Watanabe K. (2008). Common thiolation mechanism in the biosynthesis of tRNA thiouridine and sulphur-containing cofactors. *EMBO J.* 27 3267–3278. 10.1038/emboj.2008.246 19037260 PMC2609741

[B92] Shigi N. Sakaguchi Y. Suzuki T. Watanabe K. (2006a). Identification of two tRNA thiolation genes required for cell growth at extremely high temperatures. *J. Biol. Chem.* 281 14296–14306. 10.1074/jbc.M511675200 16547008

[B93] Shigi N. Suzuki T. Tamakoshi M. Oshima T. Watanabe K. (2002). Conserved bases in the TPsi C loop of tRNA are determinants for thermophile-specific 2-thiouridylation at position 54. *J. Biol. Chem.* 277 39128–39135. 10.1074/jbc.M207323200 12177072

[B94] Shigi N. Suzuki T. Terada T. Shirouzu M. Yokoyama S. Watanabe K. (2006b). Temperature-dependent biosynthesis of 2-thioribothymidine of *Thermus thermophilus* tRNA. *J. Biol. Chem.* 281 2104–2113. 10.1074/jbc.M510771200 16317006

[B95] Sierant M. Kulik K. Sochacka E. Szewczyk R. Sobczak M. Nawrot B. (2018a). Cytochrome c catalyzes the hydrogen peroxide-assisted oxidative desulfuration of 2-Thiouridines in Transfer RNAs. *Chembiochem* 19 687–695. 10.1002/cbic.201700692 29287127

[B96] Sierant M. Leszczynska G. Sadowska K. Komar P. Radzikowska-Cieciura E. Sochacka E. (2018b). *Escherichia coli* tRNA 2-selenouridine synthase (SelU) converts S2U-RNA to Se2U-RNA via S-geranylated-intermediate. *FEBS Lett.* 592 2248–2258. 10.1002/1873-3468.13124 29862510

[B97] Singer C. E. Smith G. R. (1972). Histidine regulation in *Salmonella typhimurium*. 13. Nucleotide sequence of histidine transfer ribonucleic acid. *J. Biol. Chem.* 247 2989–3000. 4337503

[B98] Sochacka E. Bartos P. Kraszewska K. Nawrot B. (2013). Desulfuration of 2-thiouridine with hydrogen peroxide in the physiological pH range 6.6-7.6 is pH-dependent and results in two distinct products. *Bioorg. Med. Chem. Lett.* 23 5803–5805. 10.1016/j.bmcl.2013.08.114 24064499

[B99] Suzuki T. Suzuki T. Wada T. Saigo K. Watanabe K. (2002). Taurine as a constituent of mitochondrial tRNAs: new insights into the functions of taurine and human mitochondrial diseases. *EMBO J.* 21 6581–6589. 10.1093/emboj/cdf656 12456664 PMC136959

[B100] Takahashi N. Wei F. Y. Watanabe S. Hirayama M. Ohuchi Y. Fujimura A. (2017). Reactive sulfur species regulate tRNA methylthiolation and contribute to insulin secretion. *Nucleic Acids Res.* 45 435–445. 10.1093/nar/gkw745 27568003 PMC5224495

[B101] Tao W. Yurkovich M. E. Wen S. Lebe K. E. Samborskyy M. Liu Y. (2016). A genomics-led approach to deciphering the mechanism of thiotetronate antibiotic biosynthesis. *Chem. Sci.* 7 376–385. 10.1039/c5sc03059e 28791099 PMC5518548

[B102] Tigano M. Ruotolo R. Dallabona C. Fontanesi F. Barrientos A. Donnini C. (2015). Elongator-dependent modification of cytoplasmic tRNALysUUU is required for mitochondrial function under stress conditions. *Nucleic Acids Res.* 43 8368–8380. 10.1093/nar/gkv765 26240381 PMC4787798

[B103] Tyagi K. Pedrioli P. G. (2015). Protein degradation and dynamic tRNA thiolation fine-tune translation at elevated temperatures. *Nucleic Acids Res.* 43 4701–4712. 10.1093/nar/gkv322 25870413 PMC4482078

[B104] Umeda N. Suzuki T. Yukawa M. Ohya Y. Shindo H. Watanabe K. (2005). Mitochondria-specific RNA-modifying enzymes responsible for the biosynthesis of the wobble base in mitochondrial tRNAs. Implications for the molecular pathogenesis of human mitochondrial diseases. *J. Biol. Chem.* 280 1613–1624. 10.1074/jbc.M409306200 15509579

[B105] Väre V. Y. Eruysal E. R. Narendran A. Sarachan K. L. Agris P. F. (2017). Chemical and conformational diversity of modified nucleosides affects tRNA structure and function. *Biomolecules* 7:E29. 10.3390/biom7010029 28300792 PMC5372741

[B106] Watanabe K. Oshima T. Saneyoshi M. Nishimura S. (1974). Replacement of ribothymidine by 5-methyl-2-thiouridine in sequence GT psi C in tRNA of an extreme thermophile. *FEBS Lett.* 43 59–63. 10.1016/0014-5793(74)81105-1 4369142

[B107] Watanabe K. Shinma M. Oshima T. Nishimura S. (1976). Heat-induced stability of tRNA from an extreme thermophile, *Thermus thermophilus*. *Biochem. Biophys. Res. Commun.* 72 1137–1144. 10.1016/S0006-291X(76)80250-1 985514

[B108] Wei F. Y. Suzuki T. Watanabe S. Kimura S. Kaitsuka T. Fujimura A. (2011). Deficit of tRNA(Lys) modification by Cdkal1 causes the development of type 2 diabetes in mice. *J. Clin. Invest.* 121 3598–3608. 10.1172/JCI58056 21841312 PMC3163968

[B109] Wittwer A. J. Tsai L. Ching W. M. Stadtman T. C. (1984). Identification and synthesis of a naturally occurring selenonucleoside in bacterial tRNAs: 5-[(methylamino)methyl]-2-selenouridine. *Biochemistry* 23 4650–4655. 10.1021/bi00315a021 6388630

[B110] Wu Y. Wei F. Y. Kawarada L. Suzuki T. Araki K. Komohara Y. (2016). Mtu1-Mediated thiouridine formation of mitochondrial tRNAs is required for mitochondrial translation and is involved in reversible infantile liver injury. *PLoS Genet.* 12:e1006355. 10.1371/journal.pgen.1006355 27689697 PMC5045200

[B111] Yokoyama S. Watanabe T. Murao K. Ishikura H. Yamaizumi Z. Nishimura S. (1985). Molecular mechanism of codon recognition by tRNA species with modified uridine in the first position of the anticodon. *Proc. Natl. Acad. Sci. U.S.A.* 82 4905–4909. 10.1073/pnas.82.15.4905 3860833 PMC390466

